# Multiple inducers and novel roles of autoantibodies against the obligatory NMDAR subunit NR1: a translational study from chronic life stress to brain injury

**DOI:** 10.1038/s41380-020-0672-1

**Published:** 2020-02-24

**Authors:** Hong Pan, Agnes A. Steixner-Kumar, Anna Seelbach, Nadine Deutsch, Anja Ronnenberg, Daniel Tapken, Nico von Ahsen, Marina Mitjans, Hans Worthmann, Ralf Trippe, Christina Klein-Schmidt, Nadine Schopf, Kristin Rentzsch, Martin Begemann, Jürgen Wienands, Winfried Stöcker, Karin Weissenborn, Michael Hollmann, Klaus-Armin Nave, Fred Lühder, Hannelore Ehrenreich

**Affiliations:** 1 Clinical Neuroscience, Max Planck Institute of Experimental Medicine, Göttingen, Germany; 2 Department of Neurology, Hannover Medical School, Hannover, Germany; 3 Department of Biochemistry I–Receptor Biochemistry, Ruhr University, Bochum, Germany; 4 Institute of Clinical Chemistry, University Medical Center Göttingen, Göttingen, Germany; 5 Institute for Experimental Immunology, Euroimmun, Lübeck, Germany; 6 Department of Psychiatry & Psychotherapy, University Medical Center Göttingen, Göttingen, Germany; 7 Institute for Cellular and Molecular Immunology, Georg August University, Göttingen, Germany; 8 Neurogenetics, Max Planck Institute of Experimental Medicine, Göttingen, Germany; 9 Institute for Neuroimmunology and Multiple Sclerosis Research, University Medical Center Göttingen, Göttingen, Germany

**Keywords:** Neuroscience, Molecular biology

## Abstract

Circulating autoantibodies (AB) of different immunoglobulin classes (IgM, IgA, and IgG), directed against the obligatory N-methyl-d-aspartate-receptor subunit NR1 (NMDAR1-AB), belong to the mammalian autoimmune repertoire, and appear with age-dependently high seroprevalence across health and disease. Upon access to the brain, they can exert NMDAR-antagonistic/ketamine-like actions. Still unanswered key questions, addressed here, are conditions of NMDAR1-AB formation/boosting, intraindividual persistence/course in serum over time, and (patho)physiological significance of NMDAR1-AB in modulating neuropsychiatric phenotypes. We demonstrate in a translational fashion from mouse to human that (1) serum NMDAR1-AB fluctuate upon long-term observation, independent of blood–brain barrier (BBB) perturbation; (2) a standardized small brain lesion in juvenile mice leads to increased NMDAR1-AB seroprevalence (IgM + IgG), together with enhanced Ig-class diversity; (3) *CTLA4* (immune-checkpoint) genotypes, previously found associated with autoimmune disease, predispose to serum NMDAR1-AB in humans; (4) finally, pursuing our prior findings of an early increase in NMDAR1-AB seroprevalence in human migrants, which implicated chronic life stress as inducer, we independently replicate these results with prospectively recruited refugee minors. Most importantly, we here provide the first experimental evidence in mice of chronic life stress promoting serum NMDAR1-AB (IgA). Strikingly, stress-induced depressive-like behavior in mice and depression/anxiety in humans are reduced in NMDAR1-AB carriers with compromised BBB where NMDAR1-AB can readily reach the brain. To conclude, NMDAR1-AB may have a role as endogenous NMDAR antagonists, formed or boosted under various circumstances, ranging from genetic predisposition to, e.g., tumors, infection, brain injury, and stress, altogether increasing over lifetime, and exerting a spectrum of possible effects, also including beneficial functions.

## Introduction

N-methyl-d-aspartate receptors (NMDAR) are abundantly expressed in mammalian brain. Acting as glutamate-gated cation channels, they form heteromers of NR1, NR2, and NR3 subunits, with NR1 as the mandatory partner (NMDAR1, *new nomenclature GluN1 disregarded here for consistency with most of the respective autoantibody literature*). NMDAR are crucial for regulating neuronal/synapse function, but are also expressed by, e.g., astrocytes, oligodendrocytes, as well as different cell types in the periphery, where their role is less understood [1–6].

NMDAR1 autoantibodies (NMDAR1-AB) of the immunoglobulin G (IgG) class in serum and CSF have originally been described as pathognomonic for “anti-NMDAR encephalitis”, characterized by psychosis, cognitive decline, dyskinesia, epileptic seizures, loss of consciousness, and autonomic instability [7–10]. As a pathophysiological mechanism, NMDAR1-AB-induced receptor internalization had been proposed [11]. Shortly thereafter, NMDAR1-AB of other Ig-classes (IgM and IgA) were also deemed relevant for neuropsychiatric phenotypes [12–17]. In vitro assays revealed similar effects of NMDAR1-AB, independent of Ig-class, on receptor internalization in human IPSC-derived and primary mouse neurons, as well as on glutamate-evoked currents in *Xenopus laevis* oocytes [17–19]. In vivo studies confirmed comparable influence of serum NMDAR1-AB of all Ig-classes on brain functions, with blood–brain barrier (BBB) permeability deciding on their pathophysiological significance [16–21].

Entirely unexpected was the demonstration of age-dependent >20% NMDAR1-AB seroprevalence in humans, including IgM, IgA, and IgG, with comparable titers and epitopes in health and disease [16–18, 21–23]. Thus, other mammals, namely, dogs, cats, rats, and mice, were screened and found age-dependently highly seropositive for functional NMDAR1-AB [20]. This age dependence was lost in baboons and rhesus macaques, i.e., non-human primates in captivity, and in human migrants, raising the intriguing possibility that NMDAR1-AB formation (predominantly of the IgA class) is related to early chronic life stress [20]. Apart from these newer observations, the occurrence of NMDAR1-AB has previously been associated with oncological conditions (teratoma) [7], influenza A/B seropositivity [17, 21], and herpes encephalitis [13]. A possible genetic predisposition was explored by a genome-wide association study, uncovering the genetic marker, rs524991, related to NMDAR biology [17].

Together, these findings indicate that naturally occurring, functional NMDAR1-AB belong to the normal autoimmune repertoire of mammals, and may have physiological roles as well as pathogenic potential, irrespective of the epitope and Ig-class [18, 20]. In the present translational work from mouse to human, we address for the first time the spontaneous intraindividual course of NMDAR1-AB in serum over time, describe novel conditions of NMDAR1-AB formation/boosting, e.g., experimental chronic life stress, and demonstrate thus far unrecognized properties of NMDAR1-AB in modulating neuropsychiatric phenotypes, i.e., ketamine-like antidepressant effects.

## Materials and methods

### Ethical approvals

Ethics Committees of Georg August University, Göttingen, and collaborating centers approved the Göttingen Research Association for Schizophrenia (extended GRAS) data collection [16, 17, 21, 22, 24, 25], Ethics Committee of Hannover Medical School permitted inclusion of stroke patients, all in agreement with Helsinki Declaration. Participants gave written informed consent. Mouse experiments were approved by the local animal care/use committee (LAVES). Experiments were performed by investigators unaware of group assignment/treatments (fully blinded).

### Human studies

#### Stroke patient follow-up

Paired blood samples of ischemic stroke patients (within 24 h after stroke and at 1–3 years follow-up; *N* = 114, 60.5% men, age at stroke 73.4 ± 11.0 [48–95] years) were collected prospectively at Hannover Medical School.

#### CTLA4 genotypes and NMDAR1-AB seropositivity

Genetic information and serology were available for 2934 subjects (63% men, age 42.9 ± 16.3 [17–95] years) of extended GRAS (*N* = 1082 schizophrenia/schizoaffective disorder, *N* = 1256 healthy, *N* = 260 Parkinson, *N* = 248 other neuropsychiatric diseases, and *N* = 88 stroke) after random exclusion of one individual/pair of second-degree relatives (PIHAT > 0.185, *N* = 83) to avoid spurious associations due to relatedness. Genotyping was performed using our semicustom Axiom-myDesign genotyping-array (Affymetrix, Santa Clara, CA, USA) described before [17, 26]. Two *CTLA4* (±5 kb flanking regions) variants, rs11571316 (MAF = 0.42) and rs3087243 (MAF = 0.46), were selected due to the highest MAF, providing maximal statistical power. Both variants were in Hardy–Weinberg equilibrium (*p* > 0.05) and strong linkage disequilibrium (*R*^2^ = 0.94).

#### NMDAR1-AB seropositivity in migrants and age-matched controls

Prospectively recruited healthy migrants (*N* = 46; 21.9 ± 4.4 [17–33] years), at the time of immigration to Germany aged 18.7 ± 4.6 years, and *N* = 821 age-matched non-migrant controls of extended GRAS were analyzed.

#### Psychopathology in *APOE4*-positive NMDAR1-AB carriers

GRAS individuals (*N* = 1046) with schizophrenia/schizoaffective disorder according to *Diagnostic and Statistical Manual of Mental Disorders (DSM-IV-TR)* and information on NMDAR1-AB serostatus, *APOE4*-carrier status [19], and *Brief Symptom Inventory* [27] items depression or anxiety were included.

### Serological analyses

#### NMDAR1-AB determination

**Fig. 1:**
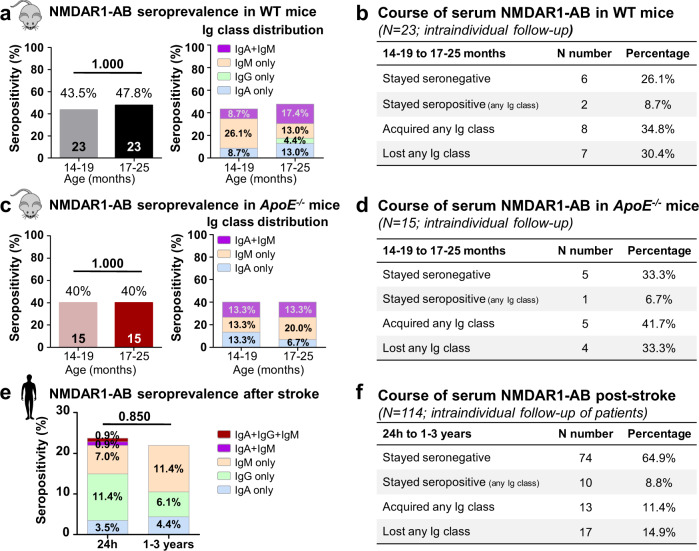
Fluctuation of NMDAR1-AB in mice and human upon longitudinal observation, independent of BBB perturbation. **a** Intraindividual comparison of NMDAR1-AB seropositivity and Ig-class distribution in aged WT mice at two different time points. **b** Course of serum NMDAR1-AB in WT mice. **c** Intraindividual comparison of NMDAR1-AB seropositivity and Ig-class distribution in aged *ApoE*^*−*^^*/−*^ mice at two different time points. **d** Course of serum NMDAR1-AB in *ApoE*^*−*^^*/−*^ mice. **e** Intraindividual follow-up of NMDAR1-AB seropositivity in stroke patients at two different time points after stroke. **f** Course of serum NMDAR1-AB in stroke patients. **a**, **c**, **e** *N* numbers/percentage displayed in bars; McNemar’s test.

**Fig. 2:**
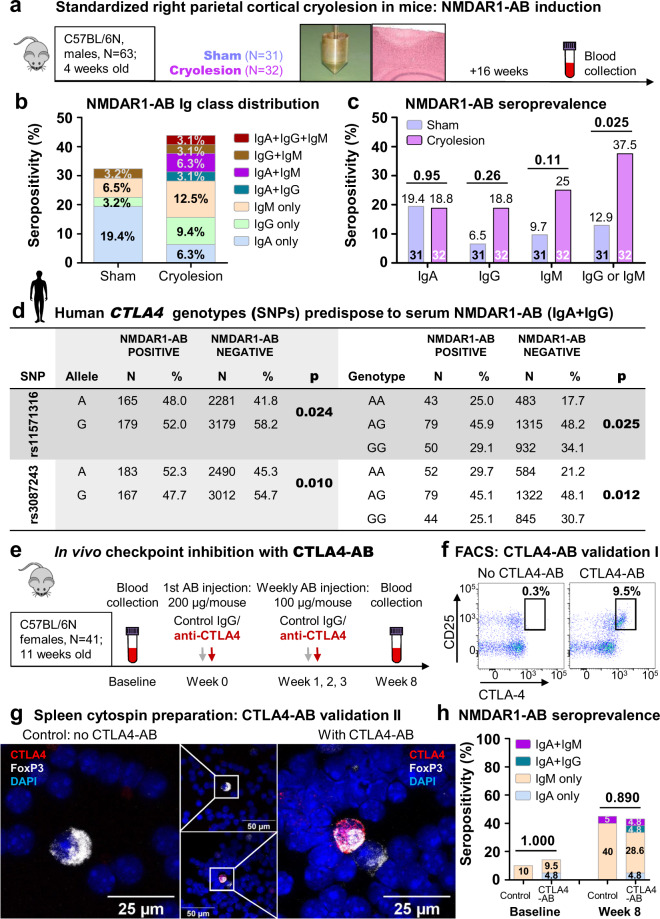
Inducers/boosters of serum NMDAR1-AB. **a** Experimental outline of the cryolesion experiment (cryolesion according to Sirén et al. [29]). **b** Higher Ig-class diversity of serum NMDAR1-AB at 4 months after cryolesion versus sham operation. **c** Increased percentage of serum NMDAR1-AB upon cryolesion is due to IgG and IgM. *N* numbers/percentage displayed in bars; chi-square test (IgA, IgM, and IgG + IgM) or Fisher’s exact test (IgG), two sided. **d** Human *CTLA4* SNPs predispose to serum NMDAR1-AB (IgA + IgG) as seen in both allelic and genotypic analyses (minor variation in *N* numbers due to missing information). **e** Experimental outline of CTLA4-AB treatment of mice. **f** CTLA4-AB validation by flow cytometry on cells from murine lymph nodes (FACS): left, control staining (without CTLA4-AB), right, CTLA4-AB staining; cells pre-gated on CD4+. **g** Representative images of FoxP3 ± CTLA4-AB staining of murine spleen cells, demonstrating specific double labeling of regulatory T-cells. **h** Intraindividual follow-up of NMDAR1-AB seropositivity upon CTLA4-AB versus isotype-control IgG treatment. *N* numbers displayed in bars; Cochran–Armitage test for trend or chi-square test, two sided.

**Fig. 3:**
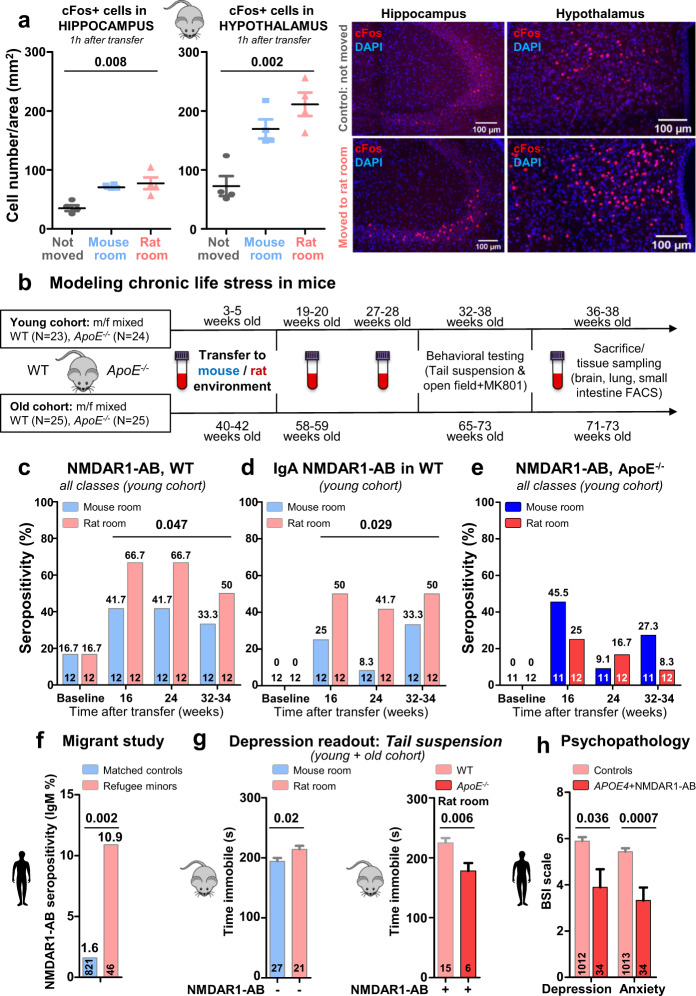
NMDAR1-AB are induced by chronic life stress and exert antidepressive effects in mice and human. **a** Pilot experiment comparatively evaluating cFos expression in mouse brain as acute stress marker at 1 h after either moving within the mouse room, or moving to the rat room, or controls, staying without moving. Left: quantification of cFos + cells in the hippocampus and hypothalamus of C57BL/6N WT males, 4 weeks old, *n* = 4/group, 2–3 sections/mouse quantified; Jonckheere’s trend test; right: representative images of cFos staining in the hippocampus and hypothalamus under control versus transfer to rat room conditions. **b** Experimental outline for modeling of chronic life stress in mice. *Young cohort*: mouse environment: *N* = 23, 11 *ApoE*^−^^*/*^^−^ (eight males, three females), 12 WT (eight males, four females); rat environment: *N* = 24, 12 *ApoE*^−^^*/*^^−^ (eight males, four females), 12 WT (four males, eight females); *old cohort*: mouse environment: *N* = 24, 12 *ApoE*^−^^*/*^^−^ (six males, six females), 12 WT (three males, nine females); rat environment: *N* = 26, 13 *ApoE*^−^^*/*^^−^ (six males, seven females), 13 WT (three males, ten females). **c** NMDAR1-AB overall seroprevalence of WT is higher in mice housed in the rat compared with the mouse room (young cohort displayed; old cohort similar—not shown). **d** This increased seroprevalence in WT is due to NMDAR1-AB of the IgA class. **e** In contrast, *ApoE*^−^^*/*^^−^ mice lack the “organized pattern” seen in WT mice over time. *N* numbers given in the bars; generalized estimating equation, one sided. **f** NMDAR1-AB seroprevalence (IgM) is higher in prospectively recruited young migrants compared with age-matched controls of the GRAS data collection; *N* numbers given in the bars; Fisher’s exact test, two sided. **g** Left: higher depressive-like behavior of seronegative mice housed in rat as compared with mouse environment (both genotypes pooled); right: comparison of seropositive mice housed in rat environment reveals an antidepressive effect of NMDAR1-AB dependent on BBB function, i.e., in *ApoE*^−^^*/*^^−^ mice. *N* numbers given in the bars; unpaired *t* test, two sided. **h** Translation to humans using the GRAS data collection: NMDAR1-AB carriers with permeable BBB (*APOE4*+) are less depressed and anxious (BSI-scale scores) compared with controls; *N* numbers given in the bars; depression, unpaired *t* test, two sided; anxiety, Welch test, two sided.

An established commercial assay, based on NMDAR1-transfected HEK293 cells (Euroimmun, Lübeck, Germany), was used to detect NMDAR1-AB in serum/plasma with the respective secondary antibodies against human (Euroimmun, Lübeck, Germany) [8, 28] or mouse IgA, IgG, or IgM (62-6700, custom-labeled with Alexa-Fluor488; A-21202; A-21042; ThermoFisher Scientific, Waltham, USA). For cryolesion experiment, an analogous noncommercial assay (HEK293T cells, mycoplasma free, Hollmann-Lab, Bochum) was used [18, 20]. The results were evaluated by three independent investigators (Figs. 1–3).

### Mouse studies

#### Right parietal cortical cryolesion model

This model was described in detail earlier [29]. In brief, 4-week-old WT C57BL/6J male mice received a standardized cryolesion using a liquid nitrogen-cooled copper cone with 1-mm-diameter tip, placed stereotactically for 60 s on the right parietal skull after scalp incision. Sham surgery was performed with the uncooled cone.

#### CTLA4-AB purification

CTLA4-AB was purified from hamster UC10-4F10-11 hybridoma (HB-304, ATCC, Manassas, USA). Cells were expanded in RPMI-1640 medium, and, upon appropriate density, cultured in PFHM-II (both ThermoFisher) for 2 weeks. Protein purification of CTLA4-AB from cell culture supernatant was conducted using 1 ml HiTrap-Protein-G High-Performance Columns (GE Healthcare, Chicago, USA). Eluted fractions were desalted using PD-10 desalting columns (GE Healthcare). CTLA4-AB was eluted in PBS and quantified using Nanodrop (Peqlab, Radnor, USA).

#### CTLA4-AB confirmation by flow cytometry

Purified CTLA4-AB was labeled with SeTau647-di-NHS (SETA-BioMedical, Urbana, USA). Labeled antibodies were separated from unconjugated dye via PD-10 desalting columns (GE Healthcare), eluted (PBS), and concentrated using Pierce Protein-Concentrators (10K, ThermoFisher). Single-cell suspension was prepared from lymph nodes (C57BL/6 mouse) and resuspended after centrifugation in FACS buffer (2% BSA, PBS). Cells were stained with following antibodies for 30 min/4 °C: CD4-PE/Cy5 (1:1000; 130312, BioLegend, San Diego, USA), CD25-biotin (1:200; 553070, BD-Biosciences, San Jose, CA, USA) plus FITC Streptavidin (1:200; 405202, BioLegend). Filtered samples were acquired on a FACSAria Sorp (BD-Biosciences), and data analyzed by FlowJo software (BD-Biosciences).

#### CTLA4 confirmation by immunocytochemistry

Single-cell suspension was prepared from spleen (C57BL/6). After washing with HBSS, cells were fixed, permeabilized, and stained with CTLA4-SeTau647 (1:100) and FoxP3-PE (1:100) antibodies (FoxP3 kit, 72-5775, ThermoFisher). Nuclei were stained with DAPI for 5 min (1:5000; D9542, Sigma-Aldrich, St. Louis, USA) at room temperature (RT). After washing twice with 1 × permeabilization buffer, cells were spotted on slides (cytospin, ROTOFIX 32A, Hettich, Kirchlengern, Germany), 1200 rpm/7 min, dried overnight, and mounted with Aqua-Poly/Mount (18606-20, Polysciences, Warrington, USA). Representative images (2048 × 2048) were taken with Leica-TCS-SP5 confocal-microscope (63 × glycerol-immersion objective, 0.5 µm intervals, Leica-Microsystems, Mannheim, Germany), then processed with FIJI-ImageJ-software (https://fiji.sc/).

#### CTLA4-AB mouse study

Female C57BL/6N mice (*N* = 41) were used (details in Fig. 2e). Based on baseline NMDAR1-AB seropositivity, mice were equally distributed into groups receiving CTLA4-AB (*N* = 21) or isotype-control IgG (*N* = 20; BE0091, Bio X Cell, West Lebanon, USA) intraperitoneally.

#### cFos immunohistochemistry

For stress pilot experiment (Fig. 3a), mice were anesthetized (Avertin, 2,2,2-Tribromoethanol; T48402, Sigma-Aldrich), transcardially perfused with Ringer (Braun-Melsungen, Germany) for 2 min and 4% formaldehyde for 10 min. Brains were 24 h post-fixed in 4% formaldehyde, cryoprotected in 30% sucrose, frozen and embedded in Tissue-Tek (4583, Sakura-Finetek-Europe, Netherlands). Coronal 30 µm-thick sections (cryostat; Leica-CM1950; Leica-Microsystems, Buffalo Grove, IL, USA) were kept in 25% ethylene glycol and 25% glycerol/PBS. Free-floating sections were blocked (1 h/RT) in 5% normal horse serum (NHS) in PBST (1 × PBS + 0.3% Triton X-100) and incubated with rabbit anti-cFos (226003; Synaptic Systems, Göttingen, Germany) 1:1000 in PBST + 5% NHS overnight/4 °C. After washing with PBS, secondary antibody donkey anti-rabbit IgG-Alexa Fluor 647 (A-31573, ThermoFisher) 1:500 in PBST + 3% NHS was incubated for 2 h/RT. Nuclei were visualized with DAPI (Sigma-Aldrich) 1:5000 for 10 min. Sections were mounted using Aqua-Poly/Mount (Polysciences). Tile scans of hippocampus/hypothalamus were acquired using the 20× air-objective from Nikon-Ti2 Eclipse (Nikon, Tokyo, Japan) and cFos+ cells counted using cell-counter-plugin of FIJI-ImageJ-software. Representative images (1024 × 1024; 1 µm intervals) were taken with Leica-TCS-SP5, then processed with FIJI-ImageJ.

#### Chronic stress study

Details of the experimental setup are given in Fig. 3. All mice were housed in standard laboratory conditions (22 ± 1 °C, 55% humidity, food/water ad libitum), and after moving kept in cages with simple top lid to allow direct contact with environment air. Blood was collected from orbital sinus at indicated time points for FACS and NMDAR1-AB determination.

#### Behavioral tests

##### Tail-suspension test

Mice were gently fixed with adhesive tape 20 mm from the tail tip, and time spent immobile recorded for 6 min with a digital camera [30].

##### Baseline and post-MK-801 locomotion in open field

this test using the non-competitive NMDAR antagonist MK-801 (M107, Sigma-Aldrich), intraperitoneally injected (0.3 µg/10 µl of PBS/g body weight), was performed as described previously [17, 20].

### Flow cytometry

#### Blood

50 µl of blood was 1:1 diluted in PBS; lymphocytes were separated using 100 µl Lymphocyte Separation Medium 1077 (C-44010, PromoCell, Heidelberg, Germany). Cells were stained for 30 min/4 °C with: CD4-PE/Cy5 (1:1000; 130312), CD8a-PE/Cy7 (1:500; 100722), B220-BV510 (1:300; 103248), Gr-1-PE (1:1000; 108408), CD11b-PerCP/Cy5.5 (1:1000; 101227), F4/80-FITC (1:1000; 123107, all from BioLegend). Filtered samples were acquired on FACSAria Sorp (BD-Biosciences), and data analyzed by FlowJo software (BD-Biosciences).

#### Lung and small intestine

Tissue was collected in RPMI-1640 containing 10% FBS on ice to maximize cell viability. Isolation/analysis of immune cells was performed according to Li et al. [31]. *T-/B-cell panel*: CD45-FITC (103108), CD45R/B220-PerCP/Cy5.5 (103235), CD138-PE (142503), CD4-APC/Cy7 (100525), CD8a-APC (100712), and Zombie Aqua (423101), all from BioLegend, 1:200 dilutions; *myeloid* *panel*: CD45-PerCP/Cy5.5 (103132), Ly6C-APC (128016), Ly6G-BV421 (127628), F4/80-FITC (123107), and Zombie NIR (423105), all from BioLegend, 1:200 dilutions, and CD11b-PE (1:200; 12-0112-81, eBioscience, San Diego, USA). Filtered samples were acquired on FACSAria-I (BD-Biosciences), data analyzed by FlowJo software (BD-Biosciences).

### Statistical analysis

Statistical analyses were performed using SPSSv.17 (IBM-Deutschland-GmbH, Munich, Germany) or Prism5 (GraphPad-Software, San Diego, California, USA). Allelic and genotypic association tests were done in PLINKv1.90 (www.cog-genomics.org/plink/1.9/) [32]. Group differences in categorical/continuous variables were assessed using Cochran–Armitage test for trend, chi-square, Fisher’s exact, McNemar’s, Mann–Whitney *U*, or Student *t* tests, dependent on data distribution/variance homogeneity, ANOVA, or generalized estimating equation employed as indicated in the figures. All *p* values are two tailed unless stated otherwise; significance threshold set to *p* < 0.05; mean ± SEM presented. Based on previous work, sample sizes (humans, mice) were selected to have statistical power to detect differences. Care was taken to use a minimum number of animals (RRR principle). Datasets were routinely screened for statistical outliers to be excluded if indicated.

## Results

### Analysis across species of the spontaneous course of NMDAR1-AB in serum reveals intraindividual fluctuations

We investigated the spontaneous intraindividual course of serum NMDAR1-AB in long-term observational studies in humans and mice. Older mice have a high probability to be seropositive [20]. Therefore, cohorts of WT and *ApoE*^−/−^ mice were screened for seroprevalence at age 14–19 months. Testing was repeated for all individuals 3–6 months after the first sampling. As illustrated in Fig. 1a–d, genotype groups showed an average of ≥40% seropositivity at both time points without considerable changes in overall Ig-class distribution. Analyzing the intraindividual course of serum NMDAR1-AB, remarkable fluctuations became obvious, comparable for both genotypes, with individual mice acquiring or losing NMDAR1-AB, others remaining seronegative or seropositive (Fig. 1b, d). Translating to older humans, a stroke population could be evaluated at 24 h post stroke and again 1–3 years thereafter. Here, a similarly undulating picture arose, with no change in absolute percentage of seropositivity or Ig-class distribution but obvious intraindividual shifts. Acquisition or loss amounted to lower overall percentages (<15%) as compared with mice (>30%) (Fig. 1e, f). Total plasma IgG, IgM, IgA, albumin, and CRP at 24 h after stroke did not differ between NMDAR1-AB carriers and non-carriers (all *p* > 0.3). In addition, we analyzed consecutive samples of non-human primates (baboons, rhesus macaques) showing equivalent fluctuations (data not shown). Together, these data across species reveal “oscillations” of serum NMDAR1-AB over time, and additionally, show that chronic BBB permeability in *ApoE*^*−/−*^ mice and poststroke patients does not seem to measurably influence serum NMDAR1-AB. The slightly (non-significantly) lower overall seropositivity in *ApoE*^*−/−*^ compared with WT mice may point to NMDAR1-AB continuously being trapped in brain [18] (see also below).

### Small cortical brain lesion in juvenile mice enhances NMDAR1-AB seroprevalence and Ig-class diversity

We next wondered whether a brain lesion at young age would induce NMDAR1-AB formation, possibly due to early accessibility of the brain to immune cells via BBB breakdown [29]. Indeed, stereotactic application of a small standardized cryolesion to the right parietal cortex of mice at age 28 days, leading to BBB leakiness as described in detail before [29], induces higher overall Ig-class diversity as well as increased NMDAR1-AB seroprevalence (IgM or IgG, but not IgA), compared with the skin-only lesion (scalp incision) of sham-operated mice (Fig. 2a–c). This is in some contrast to human stroke (an “old-age lesion”, also with compromised BBB) and perhaps explained by species differences or—more likely—the different responsiveness of the immune system at young age [33, 34]. It cannot be excluded at this point and may be interesting to explore in the future, whether lesions of other organs in young mice, e.g., the gut, would result in similar increases in NMDAR1-AB seroprevalence.

### Immune-checkpoint *CTLA4* SNPs predispose to the presence of serum NMDAR1-AB in humans

The SNPs rs3087243 (A/G) and rs11571316 (A/G) of the human *CTLA4* (cytotoxic T-lymphocyte antigen4) gene on chromosome 2q33 have been associated with susceptibility to autoimmune disease, e.g., type 1 diabetes, Graves’ disease, autoimmune hypothyroidism, systemic lupus, and Addison’s disease [35–42]. Interestingly, this allelic variation can alter regulatory T-cell frequency and the signaling threshold of CD4+ T-cells [35, 39]. We therefore asked whether also NMDAR1-AB as components of the mammalian autoimmune repertoire would be associated with these immune-checkpoint *CTLA4* variants. Indeed, we obtained significant associations upon screening of *N* = 2934 human subjects (healthy or suffering from neuropsychiatric diseases) of our GRAS database (Fig. 2d). Would this finding bring us closer to understanding autoimmune mechanisms regarding NMDAR1-AB?

### Checkpoint-inhibitor treatment (anti-CTLA4-AB) of healthy adult mice does not further enhance their already high NMDAR1-AB seroprevalence

Since treatment of cancer patients with checkpoint-inhibitors (anti-CTLA4) has led to autoimmune diseases as serious adverse events [43–46], we next treated healthy female WT mice with CTLA4-AB, starting at age 11 weeks (Fig. 2e). Whereas the CTLA4-AB used (purified from monoclonal AB-producing UC10-4F10-11 hybridoma line [47]) proved functional in lymph node FACS and spleen cytospin preparation (double labeling with CD25 or FoxP3, Fig. 2f, g), there were no increased serum NMDAR1-AB at 4 weeks after 1 month of weekly injections (week 8 after treatment start, Fig. 2h). Seropositivity, also in controls at that time point, however, was already >40%. To summarize, *CTLA4* (immune-checkpoint) SNPs and CTLA4-AB treatment, previously associated with autoimmune disease, predispose in humans, as uncovered here, also to NMDAR1-AB, while checkpoint-inhibitor treatment (CTLA4-AB) of healthy adult mice without additional immune stimulation does not further enhance their already high NMDAR1-AB seroprevalence.

### Modeling chronic life stress in WT mice leads to stress-induced enhancement of NMDAR1-AB seroprevalence mainly of the IgA class

Previously, we reported high early seroprevalence of NMDAR1-AB in non-human primates in captivity and human migrants, determined retrospectively, and interpreted these findings as a reflection of persistent life stress as potential inducer of NMDAR1-AB [20]. Searching now for a chronic stress paradigm that would not require too much interference with daily life in the cage, e.g., by handling, we developed the idea to expose mice to housing in close vicinity of their natural enemy/predator, the rat [48]. In order to evaluate the reaction of mice to this new environment, we compared the number of cFos+ cells (immediate early-gene expression as stress marker) [48] in hippocampus and hypothalamus of three subgroups of male animals, namely, mice 1 h after moving either to a rat room (cage surrounded by rat cages), or within a mouse room, or not moving at all. Figure 3a illustrates the clear stair pattern for both brain regions, with moving to the rat room reaching the highest values. We therefore chose this stress paradigm and exposed two cohorts of WT versus *ApoE*^*−/−*^ mice (young and old; groups balanced for gender), to either mouse or rat environment. Blood samples for NMDAR1-AB determinations were drawn at baseline and after 16, 24, and ~33 weeks of transfer (Fig. 3b). This prospective experimental stress study yielded in WT mice, living in rat environment, an overall increase in serum NMDAR1-AB, dominated by NMDAR1-AB of the IgA class (young cohort shown in Fig. 3c, d; old cohort similar—not shown). In contrast, the serum pattern obtained in *ApoE*^*−/−*^ mice with their leaky BBB looks “less organized” and seems not clearly interpretable, most likely due to irregular transfer to and massive trapping of NMDAR1-AB in brain (Fig. 3e) [18, 49]. This binding to brain tissue on the other hand explains the distinct behavioral effects observed in *ApoE*^*−/−*^ mice. In fact, the MK-801 open-field test resulted, as expected from our previous work [17, 20], in a clear distinction between NMDAR1-AB carriers with or without compromised BBB. Seropositive *ApoE*^*−/−*^ mice showed increased locomotion after MK-801 compared with seropositive WT, independent of environment (mouse versus rat room) and age group (young as well as old cohort). While these results indicate functionality of the NMDAR1-AB upon access to brain, immunohistochemistry did not yield differences dependent on NMDAR1-AB seropositivity regarding microglia or T-cell numbers as readouts of brain inflammation. This is less surprising when considering the complete lack of any quantifiable cellular response of that kind in the brain even upon immunization against NMDAR1 peptides, leading to extremely high circulating titers of functional NMDAR1-AB of the IgG class [20]. In addition, extensive repeated FACS of blood as well as terminal FACS of lung and gut did not reveal any considerable changes in major immune cell composition (data not shown).

### Chronic life stress in humans: replication of our previous findings of enhanced NMDAR1-AB seroprevalence in young migrants

We next tried in humans to further consolidate, in a straightforward, hypothesis-driven fashion, the stress association of NMDAR1-AB seroprevalence. This had previously been suspected for non-human primates in captivity and human migrants [20], and has now experimentally been confirmed here in mice, housed close to their natural enemies. Thus, we prospectively recruited young migrants (*N* = 46) from different countries/ethnicities (Africa/Middle East/Europe). They were 18.7 ± 4.6 years old at the time of flight as war or political refugees, many as unaccompanied refugee minors. The NMDAR1-AB seroprevalence of these refugees, determined on average 2.5 years later and compared with that of *N* = 821 age-matched individuals of the GRAS data collection without migration background, revealed again a highly significant increase. This increase consisted in this very young population still of IgM, likely before the expected class switch to IgA (Fig. 3f).

### Novel antidepressive, ketamine-like role of NMDAR1-AB upon access to the brain in humans and mice

We next wondered whether housing in a rat environment would result in a depressive-like phenotype in mice as determined by an established depression measure, the tail-suspension test [50]. Indeed, pooling all NMDAR1-AB seronegative WT and *ApoE*^−^^*/−*^ mice, and comparing individuals in mouse environment with those in rat neighborhood, showed a significant increase in immobility of the latter. Strikingly, NMDAR1-AB seropositive *ApoE*^−^^*/−*^ mice with their permeable BBB exhibited in the rat environment a clearly lower depressive-like phenotype compared with seropositive WT (Fig. 3g). Would we be able to see similar effects in humans? To address this question, we again employed deeply phenotyped subjects of the GRAS data collection. As shown in Fig. 3h, NMDAR1-AB seropositive *APOE4* carriers (*N* = 34; permeable BBB [51–53]) had significantly lower depression and anxiety ratings as compared with all controls (*N* = 1013) that do not combine both markers (APOE4+ and NMDAR1-AB+).

## Discussion

The present study addressed several yet unclear topics in the NMDAR1-AB field, which are relevant for basic and clinical research and practice, but likely also for our understanding of (patho)physiological autoimmunity beyond NMDAR1-AB.

In older subjects, mice and humans, the course of serum NMDAR1-AB fluctuates remarkably, independent of BBB intactness. Similar fluctuations have been observed previously with other autoantibodies, determined as predictors of disease probability, e.g., in type I diabetes [54]. Assuming that NMDAR1-AB are part of the normal autoimmune repertoire, the detected fluctuations might be due to just periodical boosting of the respective B cells by various possible inducers [13, 17, 21, 55], in sum adding up to the age-dependently increasing total numbers. In absence of any persistent or reappearing inducers/boosters, levels would probably rather decline over time. Another potential mechanism of fast fluctuations or rapid decrease may be the trapping of NMDAR1-AB in brain upon BBB perturbation [21], which may lead to disappearance of previously measurable serum titers. In fact, since NMDAR1-AB serum levels decreased 2 days after stroke [16], we hypothesized earlier that brain tissue with its densely expressed NMDAR1 (acutely accessible after BBB breakdown due to stroke) may “extract” circulating NMDAR1-AB [16, 21]. Indeed, we could experimentally prove in mice that the brain acts as “immunoprecipitator” [21].

Despite the well-known continued BBB leakiness after stroke, and the accessibility of immune cells to the brain, we did not find evidence of stroke to induce further serum NMDAR1-AB. This apparent lack of an effect may be due, at least in part, to stroke lesion-induced neuropathology, which often continues to progress over time from the point of the initial lesion, especially in the elderly, once again serving as an “immunoprecipitator” [21]. This could potentially veil increased amounts of NMDAR1-AB. Further stroke follow-up work will be needed to test this possibility.

In contrast to stroke (as a brain lesion of old age), a small standardized cryolesion of the right parietal cortex in juvenile mice enhanced seroprevalence and Ig-class diversity of NMDAR1-AB. Strikingly, when comparing NMDAR1-AB Ig-classes post cryolesion (physical brain damage) with those induced by chronic life stress (“only” psychological brain trauma), we see IgG/IgM prevailing in the former, IgA in the latter condition. While we already suggested an association of stress with NMDAR1-AB of the IgA class in previous work on monkeys and migrants [20], the increase in IgG/IgM (but not IgA) NMDAR1-AB seroprevalence upon brain lesion was unexpected and will require experimental and clinical follow-up studies to further confirm and explore the mechanisms underlying this highly interesting class-specific response. Since IgA is seen as “mucosal Ig”, we wondered whether chronic life stress, known to be commonly associated with an abnormal breathing pattern or with a tendency to develop diarrhea or constipation, would reveal an altered immune cell composition in lung [56] and/or gut [57] of our experimental animals. However, neither FACS of these organs nor of blood uncovered any appreciable changes in the proportion of the main immune cell subsets. Therefore, numerical alterations do not aid in explaining the here-observed Ig-class-specific response, and future work on NMDAR1-AB formation will have to explore the mechanisms prompting inducer-specific Ig-class formation.

In a first search of cellular mechanisms relevant for (patho)physiological autoimmunity in general, and NMDAR1-AB in particular, we focused here on the gene encoding *CTLA4*, an Ig-superfamily member and dampener of T-cell activation, with recognized susceptibility to various autoimmune diseases [35–42]. Correspondingly, anti-CTLA4 treatment of cancer patients can result in autoimmune disease as serious adverse event [43–46]. CTLA4 is an important regulator of the immune response, exerting its influence on reactivity to both foreign and self-antigens. Allelic variation of *CTLA4* as well as CTLA4 blockade/anti-CTLA4 treatment influences the signaling threshold of CD4 T-cells [39, 45], thereby augmenting antitumor immunity but also exacerbating/inducing autoimmune disease. Would NMDAR1-AB as components of the natural autoimmune repertoire follow the rules observed for autoimmune diseases? While we could demonstrate an association of *CTLA4* SNPs with NMDAR1-AB seroprevalence in humans, our first treatment approach in mice did not yield the expected increased NMDAR1-AB seroprevalence. This negative result may be explained by the here performed anti-CTLA4 treatment under basal housing and cage-life conditions and, accordingly, a lack of particular immune stimulation that would have led to the necessary threshold of T-cell activation. Another factor to be changed in a follow-up study might be the relatively old age of mice at treatment start with an already high percentage of NMDAR1-AB carriers at the time point of analysis.

Finally, the perhaps most intriguing finding of the present study is the antidepressive action of circulating NMDAR1-AB, induced upon experimental chronic life stress in mice, and analogously demonstrated in human NMDAR1-AB carriers. The presence of NMDAR1-AB in serum, together with a compromised BBB, allowing their access to brain, reduces depression and anxiety. The antidepressive effect of the NMDAR antagonist ketamine is well established and increasingly applied in the clinical setting [58–61]. Our across-species findings with NMDAR1-AB as “endogenous antagonists” do not only replicate in vivo functionality of NMDAR1-AB, but also raise the intriguing possibility that the body can, under certain circumstances, produce its own antidepressants. Recent work reports sustained rescue of prefrontal circuit dysfunction by ketamine-induced spine formation as potential antidepressive mechanism [62, 63]. The question of whether NMDAR1-AB as endogenous antidepressants act in a similar fashion will have to be pursued in follow-up studies, searching for further mechanistic insight.

To summarize, the present translational work demonstrates that the abundantly detected NMDAR1-AB in serum of mammals fluctuate spontaneously, are Ig-class specifically induced by brain lesion or chronic life stress, particularly at young age, and can act in an antidepressive fashion upon brain access. Building here on the highly frequent NMDAR1-AB as a convenient research tool, these findings may extend beyond NMDAR1-AB, indicate general modulatory roles of autoantibodies regarding a wide range of biological functions, and inspire a broader perspective on (patho)physiological autoimmunity.
